# PDK4 Inhibition Ameliorates Melatonin Therapy by Modulating Cerebral Metabolism and Remyelination in an EAE Demyelinating Mouse Model of Multiple Sclerosis

**DOI:** 10.3389/fimmu.2022.862316

**Published:** 2022-03-09

**Authors:** Majid Ghareghani, Zahra Farhadi, Serge Rivest, Kazem Zibara

**Affiliations:** ^1^ Neuroscience Axis, Research Center of CHU de Québec-Université Laval, Quebec City, QC, Canada; ^2^ Department of Molecular Medicine, Faculty of Medicine, Université Laval, Quebec City, QC, Canada; ^3^ Cellular and Molecular Research Center, Yasuj University of Medical Sciences, Yasuj, Iran; ^4^ PRASE and Biology Department, Faculty of Sciences-I, Lebanese University, Beirut, Lebanon

**Keywords:** melatonin, diisopropylamine dichloroacetate, experimental autoimmune encephalomyelitis (EAE), fatty acids, multiple scleorsis (MS), neuroinflammation, PDK4, PDC

## Abstract

We recently showed that melatonin ameliorates the severity of experimental autoimmune encephalomyelitis (EAE), an animal model of MS. However, efficiency of melatonin therapy was associated with side effects, manifested by slowing down of remyelination, through increasing the inhibitory effects of brain pyruvate dehydrogenase kinase-4 (PDK-4) on pyruvate dehydrogenase complex (PDC), a key enzyme in fatty acid (FA) synthesis during remyelination. In this study, we investigated the metabolic profile of FA synthesis using combination therapy of melatonin and diisopropylamine dichloroacetate (DADA), a PDK4 inhibitor, in EAE mice. Disease progression was monitored by recording the disability scores. Immunological, oligodendrogenesis and metabolic factors were also evaluated. Results showed that combination therapy of melatonin and DADA significantly reduced EAE disability scores, compared to melatonin, whereas DADA alone did not have any effect. In addition, co-therapy inhibited pro-inflammatory while increasing anti-inflammatory cytokines, significantly better than melatonin alone. Moreover, administration of combination drugs recovered the declined expression of oligodendrocytic markers in EAE, more potently than melatonin. Furthermore, co-therapy affected cerebral energy metabolism by significantly reducing lactate levels while increasing N-acetylaspartate (NAA) and 3-hydroxy-3-methyl-glutaryl-coenzyme-A reductase (HMGCR) levels. Finally, while melatonin increased lactate and PDK4 expression levels and greatly reduced PDC activity, co-therapy significantly restored PDC function while reducing the lactate levels. In summary, administration of melatonin with DADA increased the efficiency of melatonin treatment by eliminating the inhibitory effects of PDK4 on PDC’s function, a critical step for proper FA synthesis during remyelination.

## Introduction

Multiple sclerosis (MS) is a neuroinflammatory disorder, characterized by the attack of immune cells to the central nervous system (CNS) resulting in myelin and axonal damage (1). More than 2.2 million patients are suffering from MS worldwide (2). MS is revealed by demyelinated axons (plaques) whose lipid structure is produced by oligodendrocyte cells in the CNS. Failure in oligodendrocyte production leads to a pathological situation with a wide range of disabilities and paralysis, depending on the amount and severity of axonal function (3). Although the main causes of MS are still unknown, numerous factors have been proposed to affect disease progression. Among these, oxidative stress, mitochondrial dysfunction, and abnormality in fatty acid (FA) beta-oxidation have been proved to be involved in the disease state (4–6). FA synthesis within oligodendrocytes plays a key role in myelination and remyelination (7). In fact, the current immunomodulatory treatments in MS managed only to slow down the progression of the disease and to reduce the number of relapses (8). However, our group showed that FA synthesis in the remyelination process could be impaired following MS therapy, which seems to be one of the side effects of the current medications. Indeed, our previous animal study showed that while melatonin ameliorates the severity of the disease; however, its efficiency is reduced owing to its effect on FA synthesis, which is required for proper remyelination.

Melatonin is a hormone that is naturally synthesized by the pineal gland in the CNS in response to darkness as well as by the choroid plexus in circadian independent manner. Previous studies reported that melatonin increases oligodendrogenesis and modulates the function of the immune system (9, 10). For instance, it reduces the amounts of pro-inflammatory cytokines (IL-1β and TNF) and increases those of anti-inflammatory cytokines (IL-4 and IL-10) (10).

Beneficial effects of melatonin in animal models of MS made it a potential candidate for clinical investigation on MS patients. Our previous study on experimental autoimmune encephalomyelitis (EAE) animal model of MS showed that melatonin therapy ameliorates EAE symptoms. However, melatonin increased the levels of pyruvate dehydrogenase kinase-4 (PDK-4) in the brain, which inhibits the function of pyruvate dehydrogenase complex (PDC). The latter is known to be the main control point of energy metabolism which connects glycolysis to the tricarboxylic acid cycle (TCA) by producing NADH, FADH2 and subsequent oxidative phosphorylation. In addition, PDC is involved in acetyl-CoA production (11), which is a substrate for FA synthesis in the remyelination process. We showed that melatonin inhibits PDC and subsequently leads to a reduction in the substrate required for FA synthesis, which we suggested is a side effect of melatonin therapy. However, we observed that melatonin improved oligodendrogensis and modulated the immune system function. Therefore, we proposed that during remyelination, oligodendrocytes use an alternative pathway to prepare enough substrate for FA synthesis. This alternative pathway appeared to be slower than the main FA synthesis pathway that is regulated by PDC (10).

The role of melatonin at the experimental level is still controversial. Melatonin is currently tested in a clinical trial for MS patients (ClinicalTrials.gov Identifier: NCT03498131). In the current study, we aimed to investigate whether an inhibitor of PDK4, diisopropylamine dichloroacetate (DADA), would improve the efficiency of melatonin therapy by minimizing the side effects of melatonin on FA synthesis.

## Materials and Methods

### Reagent or Resource

EAE induction kit (Cat# EK-2110, Hooke Laboratories); melatonin (Cat#3550, Tocris Bioscience™); DADA (Cat#660-27-5, Tokyo Chemical Industry Co., Ltd. (TCI)); PDK4 (Cat# PA5-13776, Thermofisher); β-actin (Cat# sc-8432, Santa Cruz Biotechnology Inc); MOBP (Cat# WH0004336M1, SigmaAldrich); MBP (Cat# ab218011, Abcam); IL-1β (Cat#BMS6002, Thermofisher); TNF (Cat#BMS607-3, Thermofisher), IL-4 (Cat#BMS613, Thermofisher), IL-10 (Cat#BMS614INST, Thermofisher); Lactate (Ca#79-33-4, SigmaAldrich); N-acetylaspartate (Ca#997-55-7, SigmaAldrich).

### Animals

Adult female C57BL/6 mice (10-12 weeks old, 20-25 g) were purchased from Iran Pasteur Institute (Pasteur’s Institute, Tehran, Iran). Mice were maintained at the animal breeding center under an artificial 12:12 light:dark cycle and pathogen-free conditions. The animal experimental procedures were carried out in accordance with the protocols of the Iranian Agriculture Ministry, which conforms to the provisions of the Declaration of Helsinki (as revised in Brazil in 2013), and of the European Communities Council Directive (86/609/EEC). All experimental procedures in this study were approved by the Institutional Animal Care and Use Committee (IACUC) of Yasuj University of Medical Science (Protocol Permission number; IR.YUMS.REC.1395.2).

### Induction of EAE

EAE was induced using EAE’s induction kit, according to the manufacturers’ protocol. Briefly, the immunization solution containing the immunogenic epitope myelin oligodendrocyte glycoprotein-^35-55^ (MOG^35-55^) was emulsified with complete Freund’s adjuvant (CFA, Sigma Aldrich) and Mycobacterium tuberculosis, and was injected subcutaneously over the flank. Booster pertussis toxin (PTX, 200 ng) was injected intraperitoneally on the day of immunization and 3 days later.

### Clinical EAE Score

The weight of mice was evaluated daily, from day 7 post immunization until the end of study. In addition, mice were evaluated and scored for clinical signs of the disease from day 7 to day 23 post immunization, by at least 2 independent investigators, using 0-5 point scale (12) as follows: 0: no clinical disease, 0.5: partial tail paralysis, 1.0: complete tail paralysis or limp tail, 1.5: complete tail paralysis and partial paralysis of one hind limb, 2.0: complete tail paralysis and partial paralysis of both hind limbs, 2.5: partial paralysis of one hind limb and complete paralysis of one hind limb, 3.0: paralysis of both hind limbs without forelimb weakness, 4.0: hind limbs and one forelimb paralysis, 5.0: moribund/dead signs.

### Experimental Groups

**Figure 1:**
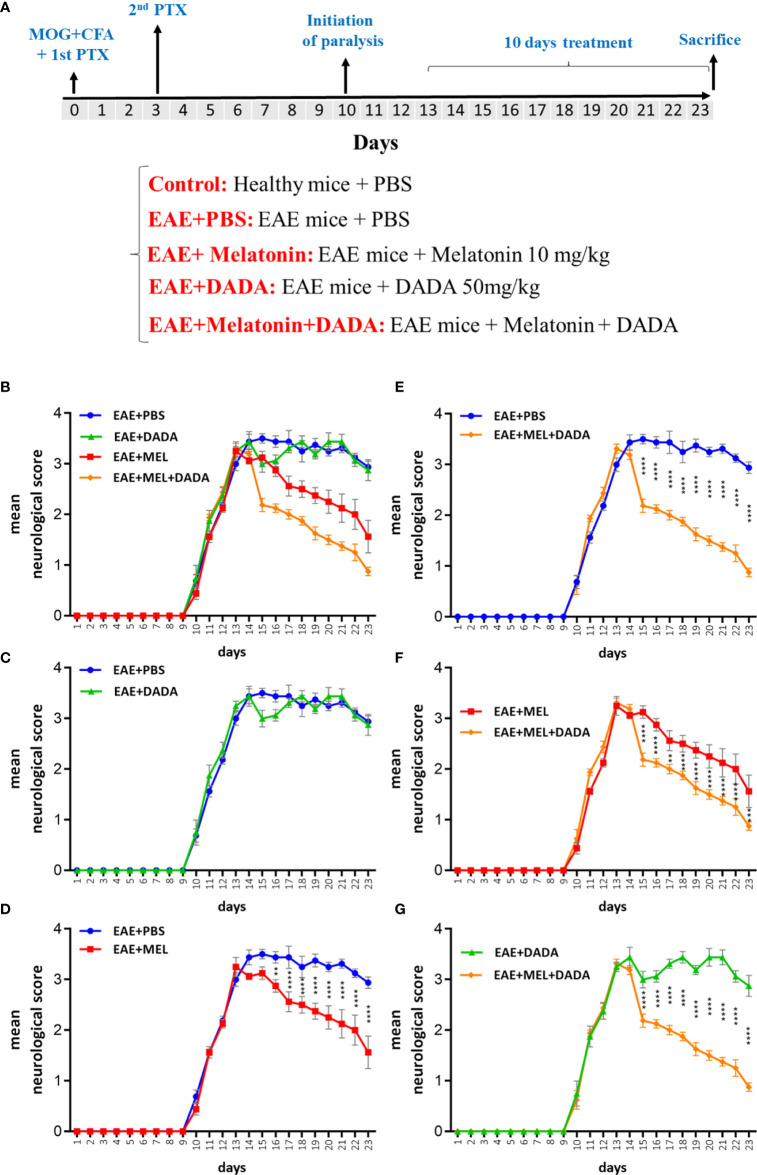
**(A)** Schematic representation of experiment procedures and **(B–G)** daily assessment of EAE severity. Neurological disability analysis displayed an amelioration in EAE disease severity in melatonin (EAE+MEL) and combined therapy of melatonin and DADA (EAE-MEL+DADA), but not DADA alone. Treatment was initiated from day 13 post immunization for 10 consecutive days. (n=8 mice/group).

A total of 32 mice were randomly divided into 4 groups of 8 mice each, as follows: (A), Control mice treated with phosphate buffered saline (Ctrl-PBS); (B) EAE mice treated with vehicle PBS (EAE-PBS); (C) EAE mice treated with melatonin at a pharmacological dose of 10 mg/kg/day (EAE-Mel); (D) EAE mice treated with a combination of melatonin (10 mg/kg/day) and DADA (50 mg/kg/day, orally) (EAE-Mel-DADA). Treatment was initiated following induction of demyelination to the same extent in each mouse. This was attained when mice reached the score of ≥ 3, which corresponds to paralysis of both hind limbs without forelimb weakness. This allowed us to study the therapeutic effect of our drugs. The mice that showed early or late diseases onset were excluded from study. Clinical scores were recorded in a manner that was blinded to mouse groups. All treatments were performed between 8:00 and 9:00 AM, when melatonin level is at its lowest. Mice were sacrificed at day 23 between 9:00 to 11:00 AM. Melatonin was freshly prepared by dissolving it in PBS and 5% dimethyl sulfoxide (DMSO). The pharmacological dose of melatonin and that of DADA were chosen based on previous studies (9, 13, 14). Control, vehicle (EAE mice) and experimental groups all received the same percentage of 5% DMSO. The experimental procedures are summarized in a schematic representation in **Figure 1A**. At the end of the study, mice were anesthetized, and brain tissues quickly excised, immediately frozen on dry ice, and stored at −80° C until further use.

### Western Blotting

Briefly, western blotting steps were carried out as follows: brain frozen tissues were homogenized on ice and lysed in a lysis buffer. The contents of the lysing buffer included 50 mM Tris – HCl (pH 7.5), 150 mM NaCl, 0.5% deoxycholic acid, 1% Nonidet P40, 0.1% SDS, 1 mM PMSF, and 100 mg/ml leupeptin. A Bio-Rad colorimetric protein assay kit (Bio-Rad, United States) was used to measure protein content. Then, proteins were separated on 8-15% SDS page. Next, gels were transferred to nitrocellulose membranes which were blocked in blocking buffer (5% skim milk solution) for 1 hour at room temperature. Then, primary antibodies were diluted in the blocking buffer and incubated at 4^°^ C overnight on a shaker. Antibody target proteins included: Pyruvate Dehydrogenase Kinase isoform 4 (PDK4, 1:400), β-actin (1:1000), Myelin-associated oligodendrocytic basic protein (MOBP; 1:400) and myelin basic protein (MBP; 1:400). After washing steps, secondary antibodies coupled with horseradish peroxidase (HRP) were added and incubated at room temperature. An enhanced chemiluminescence detection system was used for detecting proteins bound to HRP conjugated antibodies. For measuring bands density, the MyImage software was used, and then relative images quantified by image analysis software for gel documentation (LabWorks Software Version3.0, UVP Inc., United States).

### Enzyme-Linked Immunosorbent Assay (ELISA)

Brain samples were used to quantify the levels of pro-inflammatory cytokines IL-1β and TNF and anti-inflammatory cytokines IL-4 and IL-10 using ELISA kits according to the manufacturer’s instructions (R&D Systems, and Abcam, United States).

### Real-Time PCR

Quantification of PDK4 and 3-hydroxy3-methylglutaryl-coenzyme-A reductase (HMGCR) genes was performed by quantitative real-time PCR (qRT-PCR), using a StepOne Real-Time PCR system (Applied Biosystems). Reagents and supplies included RealQ Plus 2x Master Mix Green (Ampliqon, Denmark), TaqMan primer/probe assays, TRI Reagent^®^ solution (Sigma-Aldrich, the Netherlands), Sequence Detection System. To extract total RNA, Tri-Reagent was used following the manufacturer’s instructions after homogenization of brain samples. On the other hand, the High-Capacity cDNA Reverse Transcription kit (Applied Biosystems, United States) was used in accordance with manufacturer’s protocol to synthesize cDNA using random primers. Primer sequences were designed as follows: PDK4: F, CCGCTTAGTGAACACTCCTTC, and R, TCTACAAACTCT GACAGGGCTTT, HMGCR: F TGATTGGAGTTGGCACCAT, and R, TGGCCAACACTGACATGC. The specificity of PCR products was confirmed by melting curve analysis. The PCR was carried out as follows: initial activation at 95^°^C for 15 min, then 35 amplification cycles consisting of denaturation at 95^°^C for 15s, annealing at 57^°^C for 30s, and extension at 72^°^C for 30s. Data generated from the qPCR reactions were analyzed using the Comparative CT (ΔΔCT) method. All reactions were performed in triplicate using β-actin as an internal control for normalization.

### High Performance Liquid Chromatography (HPLC)

Briefly, frozen samples of brain homogenates were centrifuged for 15 min at 40,000×g and supernatants placed in an ice bath and neutralized to pH 4–5 with potassium hydroxide (KOH), and then pellets were weighed for analysis. Samples were centrifuged for another 15 min at 40,000 × g for 15 min to sediment the precipitant, potassium perchlorate (KClO4), which formed after neutralization with KOH. Supernatants were retained and filtered (0.2 µm) before lyophilization. The chromatographic measurements of brain lactate and N-acetylaspartate (NAA) were carried out with a KNAUER smartline High Performance Liquid Chromatography (HPLC) system equipped with micro vacuum degasser, LPG system, UV-VIS Detector (2550 was set at 220 nm) and a MZ ODS-C18 (250 mm × 4.6 mm, 5 µm) column. The EZCHROM elite system was used for chromatographic calculations. Determination of lactate and NAA were performed by HPLC, as previously described (Kehr, 1999; Shannon et al., 2016). The accuracy of extraction and determination of lactate and NAA in the brain were investigated using standard addition method.

### PDC Activity Determination

Pyruvate dehydrogenase complex (PDC) exists in two forms: active or dephosphorylated as well as inactive or phosphorylated froms. Inter-conversion between these forms can readily alter the flux through this complex. Both active and total PDC activities were measured based on evolution or fixation of ^14^CO_2_ [^1−14^C] on ice freeze-thawed homogenates of brains of all groups on day 23, as described previously (Johnson et al., 2001; Pliss et al., 2004, 2013). Enzymatic activity of PDC in tissue was determined. For ‘active’ PDC activity, dichloroacetate as an inhibitor of PDH kinases and sodium fluoride as an inhibitor of PDH phosphatases were used in the homogenizing buffer to preserve the phosphorylation status for active PDC activity assessment; however, purified PDH phosphatase 1 was used in the homogenates to reach the complete dephosphorylation of PDH for total activity assessment of PDC. PDC activity is expressed as munits/mg protein.

### Data Analysis

All groups were blinded to the experimenters for all the quantifications. Results are expressed as mean ± SD (standard deviation). The data distribution was analyzed by the Shapiro–Wilk normality test in addition to Brown–Forsythe which was used to check the homogeneity of variance for ANOVA test analyses. All data presented in the manuscript passed both tests and were analyzed as normally distributed and with equal variances. Descriptive and inferential statistics was applied to the data using GraphPad Prism version 6.01 (San Diego, CA, United States). P values less than 0.05 (p<0.05) were considered to be statistically significant. Significance is indicated by ∗p < 0.05; ∗∗p < 0.01; ∗∗∗p < 0.001, and∗∗∗∗p < 0.0001.

## Results

### Combination Therapy of Melatonin and DADA Ameliorated the Disability Scores of EAE Mice

The disease course in the EAE mouse model exhibits a chronic progressive-relapsing phenotype. To assess the combination therapy of melatonin and DADA on EAE severity, mice were treated by daily *i.p.* injections of melatonin at pharmacological doses (10 mg/kg/day) or DADA (50 mg/kg/day) or their combination, from day 13 post first immunization until the end of the study at day 23. Results of neurological disability scores showed lower scores in the whole experimental period for melatonin treatment (EAE+Mel) compared to vehicle group (EAE+PBS). However, DADA did not have any effect when administered alone (EAE+DADA). On the other hand, combination therapy of melatonin and DADA (EAE+Mel+DADA) considerably ameliorated and reduced EAE disability scores all the days, compared to the melatonin group alone (**Figures 1B–G**).

### Combination Therapy Modulated the Neuroinflammation

**Figure 2:**
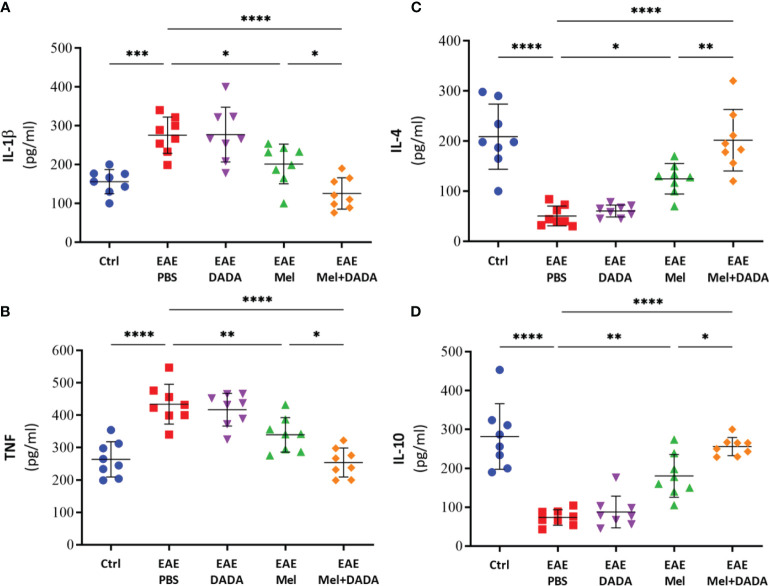
The effects of melatonin, DADA, and combination therapy on cytokine levels in the brain. Brain homogenates were used to quantify the levels of pro-inflammatory cytokines **(A)** IL-1β and **(B)** TNF and anti-inflammatory cytokines **(C)** IL-4 and **(D)** IL-10. Significance is indicated by *p < 0.05; **p < 0.01; ***p < 0.001 and ****p < 0.0001.

To investigate the role of neuroinflammation in combination therapy-induced amelioration of the disease, key cytokines of both pro-inflammatory (IL-1β and TNF) and anti-inflammatory (IL-4 and IL-10) pathways were evaluated by ELISA (**Figure 2**).. Analysis of variance showed that the effect of combination therapy was significant for IL-1β, (F (4, 35) = 15.27, p<0.000001); TNF (F (4, 35) = 19.88, p <0.000001), IL-4 (F (4, 35) = 23.814, p<0.000001), and IL-10 (F (4, 35) = 28.18, p<0.000001).

Indeed, brain levels of pro-inflammatory cytokines IL-1β (275.5 ± 46.75) and TNF (434.1 ± 61.36) were significantly (***p=0.000266 and ****p=0.000002, respectively) higher in EAE mice, compared with controls (156.1 ± 31.35 and 263.9 ± 54.31, respectively) (**Figures 2A, B**). EAE mice treated by DADA alone did not cause any effect on pro-inflammatory markers, compared to PBS treated-EAE mice, at day 23 (**Figures 2A, B**). However, melatonin therapy alone significantly reduced the expression of inflammatory cytokines of IL-1β (201.6± 51.06; *p= 0.039767) and TNF (339.5 ± 53.27; **p= 0.008897), in comparison to EAE mice. Importantly, combination therapy of melatonin and DADA significantly reduced even further the expression levels of IL-1β (125.6± 40.44; ****p=0.000007) and TNF (254.0 ± 44.55; ****p=0.000002), in comparison to the control EAE group, but also compared to melatonin alone (*p=0.032395 and *p=0.021640, respectively; **Figures 2A, B**).

On the other hand, the levels of IL-4 (50.625 ± 19.95; ****p=0.000001) and IL-10 (73.38 ± 19.99; ****p=0.000001) in untreated EAE mice were significantly decreased compared with controls (208.75 ± 69.09 and 281.9 ± 84.55, respectively) (**Figures 2C, D**). Treatment with DADA alone did not affect the expression levels of these anti-inflammatory cytokines. However, treatment with melatonin alone significantly increased the expression of IL-4 (124.88 ± 30.42; *p= 0.013284) and IL-10 (180.5 ± 54.99; **p= 0.001381), in comparison to EAE mice (**Figures 2C, D**). Importantly, combination treatment of melatonin and DADA significantly increased even further the expression levels of IL-4 (201.88 ± 61.33; ****p=0.000001) and IL-10 (256.1 ± 23.39; ****p=0.000001), in comparison to the control EAE group, but also compared to melatonin alone (**p= 0.009537 and *p= 0.037946, respectively).

In summary, these results indicate that the beneficial effect of combination treatment of melatonin and DADA in EAE mice is better than melatonin alone and is linked to an inhibition of inflammatory cytokines and an enhanced production of anti-inflammatory cytokines. Since we did not observe any effect of DADA therapy alone in EAE disability scores, inflammatory or anti-inflammatory cytokines levels, therefore, the DADA group alone was not maintained in subsequent experiments.

### Combination Therapy Potentiated the Remyelination Process

**Figure 3:**
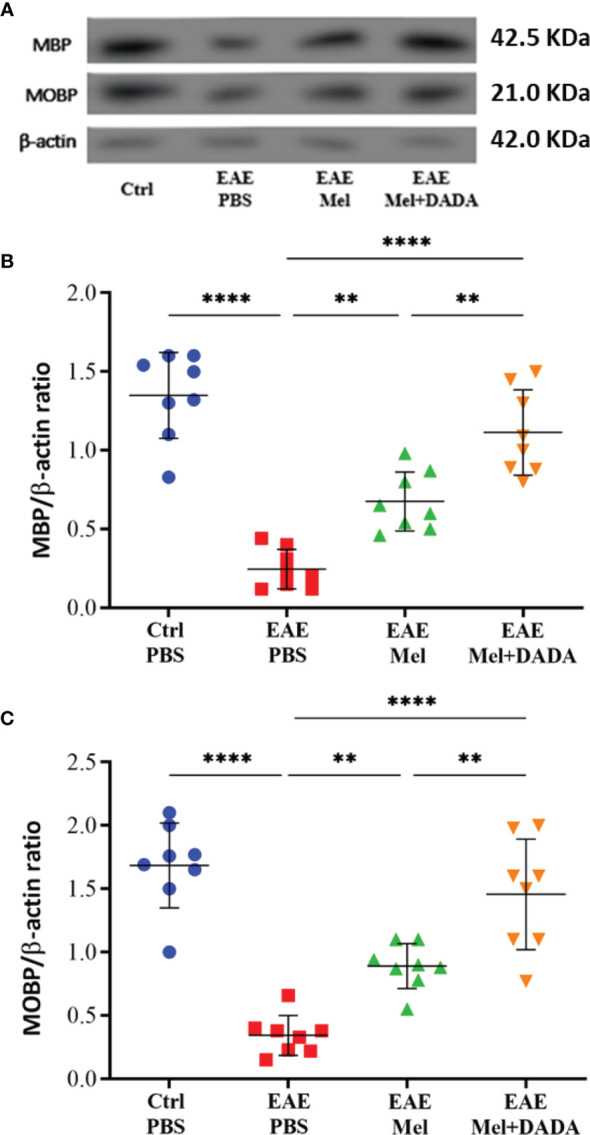
Oligodendrocyte’s expression levels. **(A)** Western blot analysis of myelin basic protein (MBP) and myelin-associated oligodendrocytic basic protein (MOBP) in the brain. The two markers (MBP and MOBP) were run alongside the same β-actin as housekeeping control. **(B, C)** Quantitative analysis for MBP and MOBP proteins, respectively. Values are expressed as the Mean ± SEM. Each group included 8 mice (n = 8). Statistical analysis was performed by one-way analysis of variance (ANOVA) followed by Tukey’s test. Significance is indicated by **p < 0.01; and ****p < 0.0001.

To test whether the combination therapy affects the protein expression levels of mature oligodendrocytic markers, brain lysates were used to perform western blot analysis on myelin basic protein (MBP) and myelin-associated oligodendrocytic basic protein (MOBP) (**Figure 3A**). One-way ANOVA analysis of variance showed the significant impact of combination therapy on MBP (F (3, 28) = 38.31, p<0.0001) and MOBP (F (3, 28) = 32.13, p<0.0001). Results showed a significant decrease in protein expression levels of MBP (0.2463 ± 0.12; ****p=<0.0001) and MOBP (0.3438 ± 0.15; ****p=<0.0001) in untreated EAE mice, compared to controls (1.349 ± 0.27 and 1.684 ± 0.33, respectively), demonstrating the loss of oligodendrocytes following EAE induction (**Figures 3B, C**). However, melatonin treatment resulted into a significant increase in protein expression levels of MBP (0.6750 ± 0.18; **p=0.0033) and MOBP (0.8900 ± 0.17; **p=0.0056), compared to untreated EAE group. Importantly, combination therapy of melatonin and DADA significantly increased protein levels of MBP (1.114 ± 0.27; ****p<0.0001) and MOBP (1.456 ± 0.43; ****p<0.0001) in comparison to EAE mice, but also in comparison to melatonin alone (**p= 0.002640 and **p= 0.004009, respectively) (**Figures 3B, C**). Therefore, administration of the combination drugs melatonin and DADA appears to induce the expression of oligodendrocytic proteins in EAE mice and to recover oligodendrocytes reduction better than melatonin treatment alone.

### Combination Therapy Modulates the Cerebral Energy Metabolism

**Figure 4:**
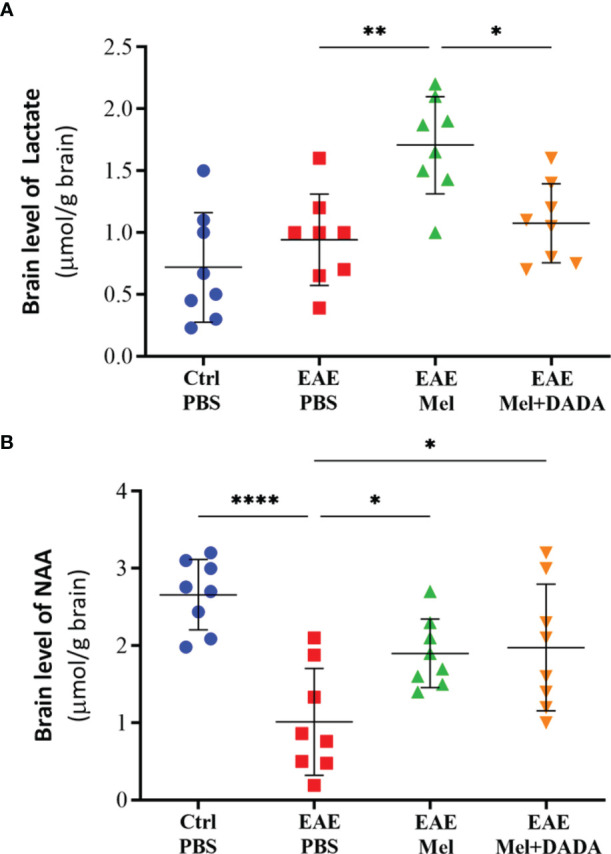
Brain levels of **(A)** lactate and **(B)** N-acetylaspartate (NAA), using HPLC. Values are expressed as the Mean ± SEM. Each group included 8 mice (n = 8). Statistical analysis was performed by two-way analysis of variance (ANOVA) followed by Tukey’s test. Significance is indicated by *p < 0.05; **p < 0.01; and ****p < 0.0001.

It has been demonstrated that lactate can be utlizied by PDC activity (15) and that NAA can be converted to acetyl-CoA in oligodendrocytes, which is required for FA synthesis in myelin (16). Therefore, we measured brain lactate and NAA, using HPLC, in order to assess the effect of combination therapy on brain metabolism and mitochondrial function which indicated the significant effect of combination therapy on lactate (F (3, 28) = 9.716, P=0.000147) and NAA (F (3, 28) = 9.400, P=0.0002). Assessment of brain lactate levels showed a rising tendency in EAE mice, compared with controls (0.9425 ± 0.37 vs 0.7188 ± 0.44), however statistical significance (p=0.653479) was not reached (**Figure 4A**). Administration of melatonin resulted in a significant increase in brain lactate concentrations, in comparison to untreated EAE mice (1.706 ± 0.39; **p<0.01) (**Figure 4A**). However, combination therapy of melatonin and DADA caused a significant reduction in lactate levels (1.075 ± 0.32; **p=0.013737), compared to melatonin treatment alone (**Figure 4A**).

On the other hand, NAA brain concentrations significantly decreased in untreated EAE mice, compared to the control group (1.013 ± 0.69 vs 2.659 ± 0.4548; ****p<0.0001, **Figure 4B**). However, melatonin treatment alone (1.900 ± 0.44; *p=0.0384) or in combination with DADA (1.975 ± 0.81; *p=0.0220) significantly increased NAA levels, compared to untreated EAE mice (*p<0.05, **Figure 4B**). Combination therapy was not better than melatonin alone in increasing NAA levels (p=0.9950). Together, these results demonstrate that combination therapy of melatonin and DADA has an effect on cerebral energy metabolism.

### Combination Therapy Restores HMGCR Expression in the Brain

**Figure 5:**
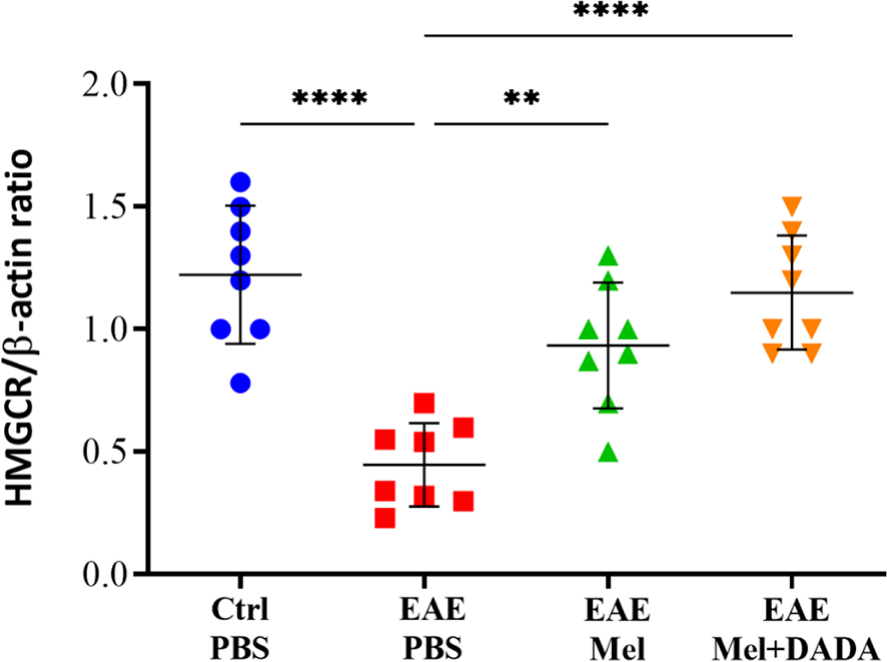
mRNA expression levels of 3-hydroxy-3-methylglutaryl-Coenzyme A reductase (HMGCR) in brain homogenates. β-actin was used as an internal control. Quantification of HMGCR expression levels was normalized to controls. Values are expressed as the Mean ± SEM. Each group included 8 mice (n = 8). Statistical analysis was performed by two-way analysis of variance (ANOVA) followed by Tukey’s test. Significance is indicated by **p < 0.01 and ****p < 0.0001

The effects of combination treatment on cholesterol biosynthesis was assessed by monitoring HMGCR enzyme activity. One-way ANOVA analysis of variance showed a significant alternation of the HMGCR (F (3, 28) = 17.13, P<0.0001). Results showed that HMGCR mRNA levels were significantly reduced in EAE mice, compared to the control group (0.4475 ± 0.17 vs 1.223 ± 0.28; ****p<0.0001) (**Figure 5**). In contrast, melatonin therapy alone (0.9338 ± 0.25) or in combination with DADA (1.150 ± 0.23) significantly increased HMGCR levels, compared with untreated EAE mice (**p=0.0019 and ****p<0.0001, respectively) (**Figure 5**).. However, the difference between melatonin (EAE+Mel) and the combination treatment (EAE+Mel+DADA) was not significant (p=0.2895), although combination therapy considerably increased the potential of melatonin in restoring HMGCR expression.

### Melatonin Therapy Increased PDK4 Expression, but Not Combination Therapy

**Figure 6:**
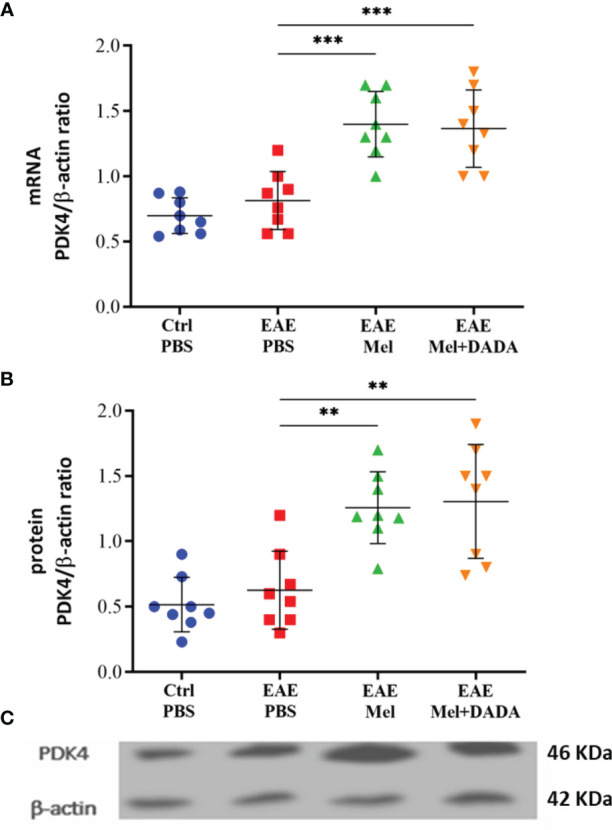
Expression of PDK4. PDK4 levels after administration of melatonin with DADA in brain homogenates **(A)** mRNA expression levels of PDK4 assessed by real time-PCR. **(B, C)** Protein expression levels of PDK4 assessed by western blot. β-actin was used as an internal control for normalization for both real time-PCR and western blot. PDK4 expression levels was normalized to controls. Significance is indicated by **p < 0.01 and ***p < 0.001.

The effect of combination therapy on PDK4 mRNA and protein expression levels was then investigated by quantitative real time-PCR and Western blot, respectively and analyzed with one-way ANOVA (mRNA level; F (3, 28) = 19.45, P<0.0001 and protein level; F (3, 28) = 19.45, P<0.0001). Induction of EAE mice did not affect PDK4 mRNA and protein expression levels compared with controls, at day 23 (p= 0.754325 and p= 0.897514) (**Figures 6A, B**). However, melatonin treatment resulted in a significant ~2-fold increase in PDK4 mRNA (1.400 ± 0.25) and protein expression levels (1.258 ± 0.27), compared with untreated EAE mice (***p=0.000156 and **p=0.002260; respectively) (**Figures 6A–C**). In contrast, combination therapy of melatonin and DADA did not have any added effect, compared to melatonin treatment alone (p=0.991453 and p=0.990333; respectively**) (** **Figures 6A, B**).

### Combination Therapy Eliminated the Inhibitory Effect of PDK4 on PDC Activity

**Figure 7:**
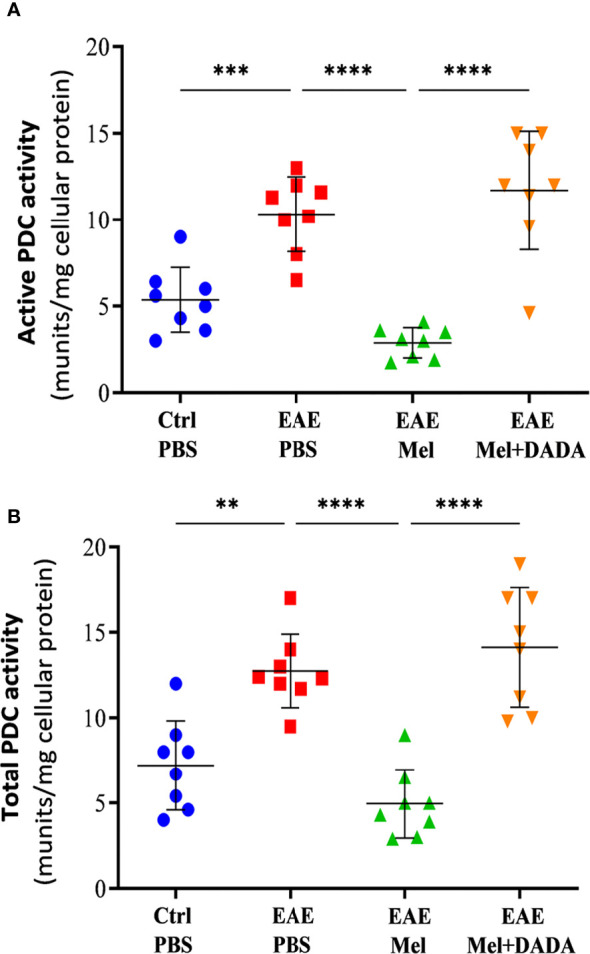
Activity of PDC. The change in the activity of PDC enzyme was measured either at the **(A)** “active” or **(B)** “total” forms of PDC. Values are expressed as the Mean ± SEM. Each group included 8 mice (n = 8). Statistical analysis was performed by one-way analysis of variance (ANOVA) followed by Tukey’s test. Significance is indicated by **p < 0.01; ***p < 0.001 and ****p < 0.0001.

Given that PDK4 controls the activity of PDC, PDC activities were assessed in both “Active” and “Total” states, in brain homogenates at day 23 using one-way ANOVA for data analysis (F (3, 28) = 26.54, P<0.0001). Results showed a significant increase (***p < 0.001) in active (10.33 ± 2.16) and total (12.74 ± 2.14) PDC activities in untreated EAE mice, compared with control group (5.363 ± 1.87 and 7.213 ± 2.62, respectively) (**Figures 7A, B**). On the other hand, this increases in PDC activity was inhibited by melatonin. Indeed, melatonin caused a significant decrease, by ~3.6 and ~2.6 –fold respectively, in active (2.83 ± 0.86) and total (4.95 ± 2.01) PDC activities, in comparison to untreated EAE mice. These inhibitory effects of melatonin were significantly abrogated by combination therapy of melatonin and DADA. Indeed, the combination treatment significantly increased active (11.70 ± 3.42; ****p<0.0001) and total (14.13 ± 3.49; ****p<0.0001) PDC activities, compared with melatonin treated EAE mice (**Figures 7A, B**).

## Discussion

In this study, we investigated the effect of combination therapy of melatonin and DADA, a PDK4 inhibitor, on the metabolic profile of FA synthesis in EAE mice. Our results showed that combination therapy significantly reduced EAE disability scores, compared to melatonin, whereas DADA alone did not have any effect. In addition, co-therapy decreased pro-inflammatory while increasing anti-inflammatory cytokines, significantly better than melatonin alone. Moreover, combination drugs recovered the expression of oligodendrocytic markers, more potently than melatonin. Furthermore, co-therapy affected cerebral energy metabolism by significantly reducing lactate levels while increasing NAA and HMGCR levels. Finally, co-therapy significantly restored PDC function while reducing the lactate levels in comparison to melatonin which increased lactate and PDK4 levels while reducing PDC activity. In summary, administration of melatonin with DADA increased the efficiency of melatonin treatment by eliminating the inhibitory effects of PDK4 on PDC function, a critical step for proper FA synthesis during remyelination.

The immune system is influenced by melatonin, a master circadian rhythm hormone in the body. However, the effects of melatonin are sometimes contradictory since they depend on several factors including age, sex, species of animals, time and method of melatonin administration, and various stressor factors. For instance, while most studies on animal models of MS reported that melatonin modulated the immune system and improved oligodendrogenesis (10, 17–26), our previous study on Lewis rats showed an age-dependent effect of melatonin in EAE treatment since treating young EAE rats with melatonin (10 mg/kg/d) exacerbated the severity of EAE. However, as a drawback of the study, melatonin was administered for a short period of 8 subsequent days (27). In fact, longer treatment periods and different ages are suggested for better understanding of age-dependent effect of melatonin. Consistent with this observation, another study showed that inhibition of the direct effects of melatonin on cells, using Luzindole as an antagonist of melatonin receptors, suppressed EAE development after immunization. Therefore, it has been proposed that the immune-enhancing effects of melatonin in demyelination may be suppressed by inhibition of melatonin receptors (28). In the current study, we showed the beneficial role of melatonin in immune cell modulation and in increasing the remyelination process. Indeed, treatment of EAE mice by melatonin resulted into a significant reduction in pro-inflammatory cytokines IL-1β and TNF and a significant increase in anti-inflammatory cytokines IL-4 and IL-10, which was associated with elevation in protein levels of oligodendrocyte markers MBP and MOBP.

On the other hand, the current study showed that melatonin treatment affects the key enzymes involved in FA synthesis and required for proper myelin synthesis. Although no significant difference in PDK4 expression level was observed following EAE induction, the activity of PDC increased significantly, which reflects the natural potential of mice brain in pathological conditions of myelin loss for self-regeneration. This increase in PDC was impaired by melatonin (**Figure 7**). Indeed, melatonin administration increased the PDK4 levels both at the transcriptional and translational levels in EAE mice while reducing the PDC activity (**Figures 6** and **7**). This observation was in line with our previous study (10), and that of Sharman et al. who also reported an increase in PDK4 mRNA levels in the CNS of mice following melatonin treatment (29). We suggest that this alteration in PDK4 level and PDC activity is in fact a side effect of melatonin therapy.

On the other hand, we previously measured the level of lactate in the brain of EAE Lewis rats and we proposed lactate as a potential biomarker in the diagnosis of MS progression (27). However, lactate levels in EAE mice in the current study were higher than control mice, although not statistically significant. It is noteworthy that a clinical study on MS patients already showed that serum lactate levels is 2.8 times higher in MS than controls (30). While melatonin exacerbated the severity of monophasic EAE in young Lewis rats, highlighting the age dependent action of melatonin, this exacerbation in EAE symptoms was associated with an increase in lactate levels (27). Since boosting PDC activity leads to a reduction in lactate accumulation (15), this study demonstrated that a reduction in PDC activity, caused by an increase in PDK levels following melatonin therapy, was accompanied by accumulation of lactate in the brain. Considering the fact that pyruvate in oligodendrocytes, obtained from the blood or from lactate and glucose conversion, needs first to be converted to acetyl-CoA (31), we hypothesized that inhibition of PDC, because of PDK elevation, limits the pyruvate conversion to acetyl-CoA, which leads to the accumulation of pyruvate (10). This acetyl-CoA is required to produce metabolites for FA synthesis. While it is already suggested that both aerobic and anaerobic glycolysis convert pyruvate to lactate (32), this could explain why PDC inhibition by melatonin increases the lactate levels.

In addition to the role of PDK and PDC in FA synthesis, cholesterol is another factor that is located in myelin sheaths and cell membranes of neurons and astrocytes (33). The synthesis of cholesterol is regulated by the key enzyme HMGCR. It is already suggested that cholesterol synthesis is limited during demyelination; however, its synthesis is upregulated during remyelination as it is required for myelin synthesis (34). Investigations on EAE and lysolecithin demyelination models showed that HMGCR expression is downregulated at the peak of the disease (34–36), and that this is associated with a reduction in the expression of myelin proteins (35). Consistent with these observations, we demonstrated that while HMGCR expression is reduced in EAE mice, compared to controls, it was correlated with demyelination, as reported by quantifying the expression of oligodendrocyte markers MBP and MOBP. In addition, we observed that melatonin restored HMGCR expression as well as MBP and MOBP. This suggests that increased HMGCR expression is an indicator of ongoing remyelination which needs more cholesterol to be synthesized by HMGCR.

Although melatonin reduced the activity of PDC, which is required for proper and efficient FA synthesis during remyelination; however, it still improved EAE severity. We considered this suppressive effect of melatonin on PDC as a side effect of melatonin, caused by using a pharmacological dose of melatonin; hence, we tried to minimize this side effect in the EAE model by combination therapy with DADA. DADA, also called “Vitamin B15” is the active component of many formulations of pangamic acid (37). DADA acts as a safe inhibitor of PDK4 and its affinity to PDK4 is 12.5-fold more than other isoforms of PDK’s such as PDK2 (14). There are limited studies regarding DADA effects in the body. An experimental study in a severe influenza model in mice showed the high potential of DADA in reducing the cytokine storm including IL-2, IL-6, IFN-*α*, TNF, and IFN-*γ*, in addition to its role in restoring the down-regulated PDH activity caused by influenza (14).

In the current study, we observed no difference between EAE mice and DADA treated EAE mice regarding clinical scores and cytokines expression. The most possible explanation for this observation could be the unchanged level of PDK4 in EAE mice, which does not involve the PDK4 modulation as a potential factor for treatment. However, melatonin increased the PDK4 level, which highlights the possible effect of PDK4 modulation in EAE progression following melatonin therapy. Indeed, DADA potentiated the beneficial effects of melatonin on reducing the pro-inflammatory cytokines (IL-1β and TNF) and increasing the anti-inflammatory cytokines (IL-4 and IL-10). In fact, DADA therapy alone would not be a potential therapeutic candidate for ameliorating EAE symptoms. DADA improved the potential of melatonin and increased oligodendrogenesis which was assayed by protein expressions of MBP and MOBP. Lactate levels, but not NAA and HMGCR, were increased by melatonin treatment, however, they were reduced after combination therapy with melatonin and DADA.

On the other hand, NAA, a nervous system-specific metabolite (38) whose role in remyelination has not been fully elucidated yet, is known to get transferred into oligodendrocytes, converted to acetate and then acetyl-CoA, a substrate for FA and cholesterol synthesis. This seems to be the alternative pathway that is potentiated following the decrease in PDC activity. This alternative pathway appeared to be slower than the main FA synthesis pathway regulated by PDC. Given that the measurement of axonal injury can be carried out by magnetic resonance spectroscopy of NAA for quantification of the resonance intensity of a neuronal marker (39), we showed here that NAA is significantly decreased flowing EAE induction. Similarly, previous studies reported a reduction of brain NAA in MS and which precedes neuronal atrophy (38, 40). Although melatonin slowed down the main pathway of FA synthesis by inhibition of PDC activity, leading to lower levels of acetyl-CoA synthesis required for FA synthesis; however, melatonin caused an increase in NAA, which is another source of acetyl-CoA production mainly exported from neuron to oligodendrocytes. This can partly explain why melatonin still increases the remyelination process despite its inhibitory role in PDC activity. However, adding DADA to melatonin therapy did not boost NAA levels. Therapeutically, it is reported that treatments of MS patients with interferon beta-1b, fluoxetine, and glatiramer acetate were associated with partial recovery of NAA levels (39, 41, 42).

Although there is a limited number of clinical studies regarding PDK/PDC axis in MS patients, Nijland and colleagues’ demonstrated increased expression of axonal PDC in demyelinated lesions of MS patients and suggested that glucose metabolism is increased in axonal lesions (43). Consistent with these data, we showed an increased PDC activity following EAE induction.

In the current study, we examined the alternation of many factors, especially PDK4 and PDC, in whole brain homogenates. However, to uncover the exact source of PDK4 and PDC changes, it would be crucial to investigate these factors in specific cell populations including oligodendrocytes, microglia, and astrocytes. Interestingly, while the main pathway of acetyl-CoA production in oligodendrocytes was thought to be PDC, whose function is under the control of PDK-4, a study using impairment of PDC activity in oligodendrocytes showed that inhibition of acetyl-CoA production through the PDC pathway is not necessary for myelin maintenance (44). One limitation of the current study is the need to assess PDC when cells require more acetyl-CoA for synthesis of the new myelin sheets, which necessitates to investigate PDC in pathological conditions of demyelination. Furthermore, the source of acetyl-CoA used by oligodendrocytes for maintenance of myelin was not explored. In fact, high amounts of fatty acids could be provided by oligodendrocytes or dietary sources, in cases of deficiency of fluxed fatty acids from astrocytes (45).

## Conclusion

**Figure 8:**
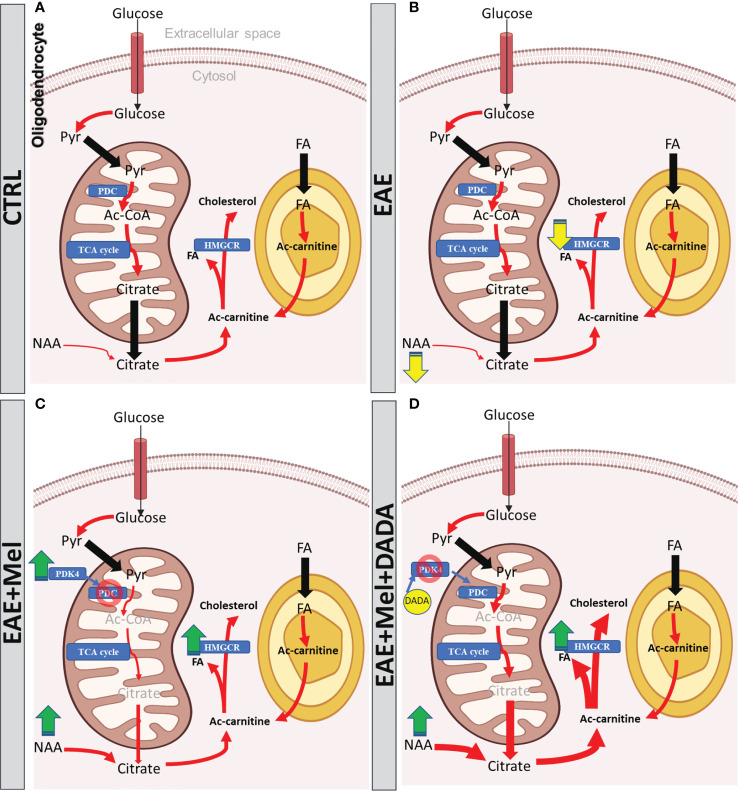
The models of Melatonin and DADA effects on FA synthesis during remyelination. **(A)** In physiological conditions, citrate is produced from imported glucose or pyruvate and from imported NAA into the oligodendrocyte to be utilized as a substrate for FA synthesis required from myelination. Proper function of PDC warranty the synthesis of this citrate. **(B)** During EAE, PDC function remains intact, which is involved in pyruvate conversion into citrate. The other precursor of citrate, NAA, shows significant reduction, which is suggested to reduce the citrate level, which might slow down FA synthesis and subsequently the remyelination in EAE mice. **(C)** Melatonin therapy recovers the reduced level of NAA in EAE mice in order to increase the citrate levels required for FA synthesis. On the other hand, melatonin increases PDK4 expression which acts as an inhibitor of PDC, leading to suppression of citrate synthesis. However, the oligodendrocyte still imports the NAA from axons or any other unknown sources to have enough citrate for FA synthesis. **(D)** Since PDC inhibition by melatonin could be considered as a side effect of melatonin therapy, DADA administered in combination with melatonin would suppress the function of PDK4 and rescue the PDC from inhibiting PDK4 function. This leads to the recovery of citrate synthesis by the PDC-dependent pathway, while NAA, another source of citrate, is already high due to melatonin, resulting together into the fast formation of sufficient sources for FA synthesis in remyelination. Yellow arrow shows reduction, green arrow shows increase, red arrow shows conversions; blue rectangle shows the enzymes, Pyr, pyruvate; FA, Fatty acid; PDK-4, pyruvate dehydrogenase kinase-4; PDC, pyruvate dehydrogenase complex;, Ac-CoA, acetyl-CoA; HMGCR, 3-hydroxy3-methylglutaryl-coenzyme-A reductase; TCA, tricarboxylic acid cycle; NAA, N-acetylaspartate.

Although melatonin therapy ameliorates the severity of EAE by modulating the immune system function and by increasing oligodendrogenesis, it reduces the activity of PDC through PDK4 elevation, which seems to slow down the FA synthesis required for re-myelination in EAE. This inhibition on PDC activity by melatonin was eliminated by combination therapy with DADA, which inhibits the PDK4 activity and hence does not allow PDK4 to inhibit PDC. This potentiated the beneficial role of melatonin in EAE therapy. The main findings of this study has been summarized as schematic models in **Figure 8**. To date, no clinical studies in MS patients have investigated the PDK/PDC axis in lesions of different MS types. Further experimental and clinical studies are required to elucidate the function of this axis in MS pathogenesis in the aim of finding a new therapeutic strategy.

## Data Availability Statement

The original contributions presented in the study are included in the article/**Supplementary Material**. Further inquiries can be directed to the corresponding authors.

## Ethics Statement

All experimental procedures were approved by the Institutional Animal Care and Use Committee (IACUC) of Yasuj University of Medical Science.

## Author Contributions

All authors contributed to the acquisition and analysis of data. MG, KZ, and SR contributed to the conception, design of the study, writing-original draft, and writing-review and editing. All authors contributed to the article and approved the submitted version.

## Funding

This work was supported by a grant from Lebanese University (KZ).

## Conflict of Interest

The authors declare that the research was conducted in the absence of any commercial or financial relationships that could be construed as a potential conflict of interest.

## Publisher’s Note

All claims expressed in this article are solely those of the authors and do not necessarily represent those of their affiliated organizations, or those of the publisher, the editors and the reviewers. Any product that may be evaluated in this article, or claim that may be made by its manufacturer, is not guaranteed or endorsed by the publisher.
