# Supplementary figures and images for: PDK4 Inhibition Ameliorates Melatonin Therapy by Modulating Cerebral Metabolism and Remyelination in an EAE Demyelinating Mouse Model of Multiple Sclerosis
Source: Front Immunol. 2022 Mar 9;13:862316. doi: 10.3389/fimmu.2022.862316 (PMC8959827; doi:10.3389/fimmu.2022.862316)

## Slide 1
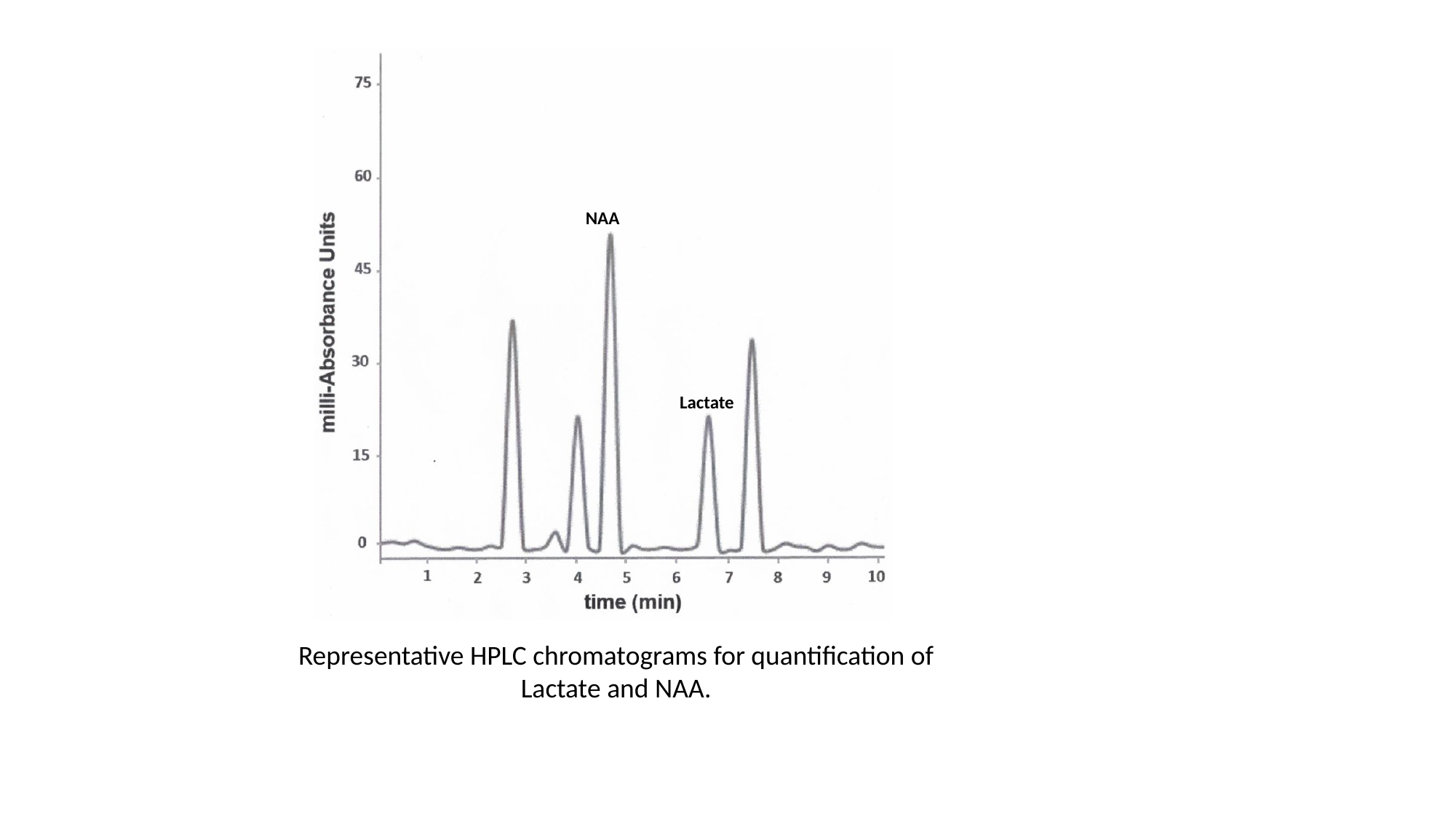

NAA
Lactate
Representative HPLC chromatograms for quantification of Lactate and NAA.

Supplement: Supplementary file 1 [file Presentation_1.pptx]
